# Study on the Effect and Mechanism of the Outer Membrane Vesicles of *Porphyromonas gingivalis* on the Function and Extracellular Matrix of Mouse Aortic Smooth Muscle Cells

**DOI:** 10.3390/microorganisms14061184

**Published:** 2026-05-24

**Authors:** Hongqiao Li, Chenyu Liu, Yan Tang, Zongmei Chen, Song Ge

**Affiliations:** School and Hospital of Stomatology, Zunyi Medical University, Zunyi 563000, China; lhqiao0308@163.com (H.L.); chenyu_liu6281@163.com (C.L.);

**Keywords:** *Porphyromonas gingivalis*, outer membrane vesicles, vascular smooth muscle cells, cytoskeleton-associated protein 4, atherosclerosis, extracellular matrix

## Abstract

Periodontitis is closely linked to atherosclerosis; however, the role of the keystone periodontal pathogen *Porphyromonas gingivalis (P. gingivalis)*, particularly its virulence factor, outer membrane vesicles (OMVs), in vascular smooth muscle cell (VSMC) dysfunction remains unclear. This study aimed to explore the effects of *P. g*-OMVs on mouse aortic smooth muscle cells (MOVAS) and the potential involvement of cytoskeleton-associated protein 4 (CKAP4) in this process. OMVs were isolated by ultracentrifugation and characterized by transmission electron microscopy (TEM), nanoparticle tracking analysis (NTA), and Western blotting. MOVAS cells were treated with OMVs; cellular functions were evaluated using CCK-8, colony formation, scratch wound-healing, ELISA, and Western blotting assays. Lentiviral vectors were used to construct CKAP4 overexpression and knockout cell models. Results showed that after *P. g*-OMVs were internalized by MOVAS cells, the cells showed cytoskeletal disorganization, promoting cell proliferation, wound closure, and contractile-to-synthetic phenotypic switching (decreased α-SMA and increased OPN expression), and enhancing extracellular matrix (ECM) remodeling (upregulated expression of type I collagen, type III collagen, fibronectin, matrix metalloproteinase-2 and -9, and tissue inhibitor of metalloproteinase-1). At the protein level, *P. g*-OMV treatment was associated with upregulated expression of CKAP4, integrin α5, and integrin β1; CKAP4 overexpression synergized with OMV stimulation to amplify these phenotypic alterations, whereas CKAP4 knockout attenuated these cellular changes. These findings suggest an association between CKAP4 upregulation and *P. g*-OMV-induced MOVAS dysfunction, indicating that CKAP4 may serve as a potential target in periodontitis-associated atherosclerosis.

## 1. Introduction

Cardiovascular diseases (CVDs) remain the leading cause of mortality and a major global socioeconomic burden. According to the World Health Organization, approximately 17.9 million deaths each year are attributable to CVDs, accounting for 32% of all deaths worldwide. Atherosclerosis, the pathological basis of most cardiovascular events, is a chronic inflammatory disorder characterized by multiple pathological processes, including lipid accumulation, infiltration of monocytes and macrophages, excessive proliferation and migration of vascular smooth muscle cells (VSMCs), and dysregulated extracellular matrix (ECM) metabolism [1]. Despite the well-established roles of traditional risk factors such as hypertension, diabetes, hyperlipidemia, and smoking, the molecular pathogenesis of atherosclerosis remains incompletely understood, highlighting the urgent need to identify novel, targetable risk factors for prevention and treatment [2].

The oral cavity hosts a complex microbial ecosystem organized as biofilms, where microorganisms are encased within a three-dimensional extracellular polymeric matrix and adhere to tooth surfaces via physicochemical interactions. Within these microbial communities, distinct biological niches sustain unique metabolic activities, and the consequent metabolic shifts disrupt the symbiotic homeostasis, thereby leading to dysbiosis and the overgrowth of pathogenic species such as *Porphyromonas gingivalis* (*P. gingivalis*) [3]. Recently, chronic infectious inflammation has been recognized as an important contributor to atherogenesis. Periodontitis, a chronic infectious disease driven by dysbiotic dental plaque microbiota, is epidemiologically associated with atherosclerosis and related CVDs independently of conventional risk factors [4]. As the primary etiological pathogen of periodontitis, *P. gingivalis* and its virulence factors have been repeatedly identified in human atherosclerotic plaques [5,6]. However, despite these observational findings, the exact causal relationship between periodontitis and atherosclerosis remains uncertain. Clinical evidence indicates that antibiotic treatment fails to reduce adverse cardiovascular outcomes in patients with both periodontitis and CVDs, and viable *P. gingivalis* is rarely isolated from atherosclerosis lesions [7]. These findings suggest that *P. gingivalis* may promote vascular dysfunction via remote-acting mechanisms, yet the molecular basis of this remote action is largely unknown.

Outer membrane vesicles (OMVs) are nanosized lipid bilayer structures secreted by Gram-negative bacteria. They contain a concentrated cargo of virulence factors, including lipopolysaccharide (LPS), gingipains, and small RNAs [8,9]. *P. g*-OMVs can cross tissue barriers, enter the systemic circulation, and target distant organs [10]. Although recent studies suggest *P. g*-OMVs may act as remote mediators of vascular injury by impairing endothelial integrity [11,12]. Nevertheless, the majority of existing evidence derives from endothelial-focused or acute inflammatory models, and the direct impact of *P. g*-OMVs on VSMC behavior within the atherosclerotic plaque microenvironment has yet to be clearly clarified. It is worth noting that endothelial damage is merely the starting trigger of atherosclerosis. During atherosclerotic plaque progression, phenotypic switching and disordered ECM remodeling of VSMCs act as key drivers of plaque development and stability [13].

VSMCs serve as the main cellular component of the arterial media. Under physiological conditions, VSMCs maintain a quiescent contractile phenotype that preserves vascular tone and wall integrity, which is distinguished by high expression of α-smooth muscle actin (α-SMA) [14]. When challenged with pathological stimuli, VSMCs undergo phenotypic switching and transform into a synthetic phenotype, acquiring potent capacities for proliferation, migration, and ECM synthesis. While this response supports tissue repair, its sustained activation in atherosclerosis promotes fibrous cap formation and plaque remodeling. This pathological change is characterized by decreased α-SMA expression and elevated levels of osteopontin (OPN) and matrix metalloproteinases (MMPs) [15], along with markedly enhanced VSMC proliferation, migration, and ECM secretion [16].

While it is well documented that *P. g*-OMVs can activate host cells through pattern-recognition receptors, including Toll-like receptor 4 (TLR4) [17], TLR-mediated signaling mainly drives acute inflammatory responses and cannot fully explain why VSMCs undergo sustained phenotypic switching and ECM remodeling during plaque progression [18]. These biological processes rely on cytoskeletal reorganization and mechanotransduction [19], with integrin α5β1 (ITGα5β1) serving as a critical sensor of ECM stiffness [20].

Cytoskeleton-associated protein 4 (CKAP4), also known as CLIMP-63, is a type II transmembrane protein that regulates microtubule binding and sustains the structural integrity of the endoplasmic reticulum [21]. Emerging evidence indicates that CKAP4, which is also expressed on the plasma membrane, regulates cell proliferation, migration, and signal transduction [22,23]. CKAP4 regulates the proliferation-apoptosis balance of VSMCs and is regulated by the CDR1as/miR-7 axis [24]. Additionally, evidence suggests that CKAP4 interacts with integrin β1 to modulate integrin α5β1 recycling, thereby regulating cell adhesion and migration [25]. ITGα5β1 acts as the major receptor for collagen and fibronectin in VSMCs, and is indispensable for sensing ECM stiffness and driving cytoskeletal reorganization [26]. While these findings suggest that CKAP4 contributes to VSMC homeostasis, it remains unclear whether CKAP4 participates in VSMC dysfunction induced by *P. g*-OMVs, and its potential role in periodontal pathogen-associated atherosclerosis also remains to be determined.

Based on the aforementioned evidence, this research was designed to address two interrelated questions: whether *P. g*-OMVs directly affect VSMC function and ECM metabolism; and whether CKAP4 is involved in mediating these OMV-induced changes. Accordingly, we hypothesized that *P. g*-OMVs may be internalized by VSMCs and thereby upregulate CKAP4 expression, which could subsequently activate integrin α5β1 signaling, potentially promoting phenotypic switching in VSMCs from a contractile to a synthetic phenotype, enhancing proliferation and migration, and accelerating ECM deposition and remodeling, and thus possibly exacerbating atherosclerosis progression. To test this hypothesis, we used mouse aortic smooth muscle cells (MOVAS) as an in vitro model. Optimal *P. g*-OMV concentration was determined by gradient screening. VSMC functions and ECM-related indicators were evaluated using CCK-8, colony formation, scratch wound-healing, ELISA, and Western blot assays. Furthermore, lentivirus-mediated CKAP4 overexpression and CRISPR/Cas9-mediated CKAP4 knockout were performed to test whether CKAP4 is involved in the remodeling phenotype observed following *P. g*-OMV exposure. If supported by our experimental findings, this study may provide preliminary correlative evidence for periodontal pathogen-driven atherogenesis and support the development of early interventions and targeted strategies for CVDs.

## 2. Materials and Methods

### 2.1. Isolation of P. g-OMVs

*P. gingivalis* (ATCC 33277, BNCC, Beijing, China) was cultured in brain heart infusion (BHI) broth (Cat No: 1405551, Coolaber, Beijing, China) supplemented with 0.5% hemin chloride (Cat No: H8132, Solarbio, Beijing, China) and 0.1% menadione (Cat No: V8151, Solarbio) at 37 °C under strictly anaerobic conditions (80% N_2_, 10% H_2_, 10% CO_2_) in an anaerobic incubator. After 6–7 days of enrichment culture, the fourth bacterial passage was harvested at an optical density at 600 nm (OD_600_) of approximately 1.0, which had been previously calibrated to correspond to 1.0 × 10^8^ colony-forming units (CFU)/mL via serial ten-fold dilutions and colony counting on blood agar plates supplemented with 0.1% menadione.

Bacterial culture supernatants were collected by centrifugation at 8000× *g* for 30 min at 4 °C, followed by filtration through 0.22 μm sterile filters to eliminate residual bacteria and debris. The filtrate was concentrated using 100 kDa molecular weight cut-off ultrafiltration centrifugal tubes (Cat No: UFC910024, Millipore, Burlington, MA, USA) by centrifugation at 2500× *g* for 30 min at 4 °C, and the concentrated volume was recorded.

The concentrated solution was subsequently subjected to ultracentrifugation at 100,000× *g* for 80 min at 4 °C using an ultracentrifuge. The resulting pellet was gently resuspended in PBS (Cat No: G0002, Servicebio, Wuhan, China), filtered again through 0.22 μm sterile filters to ensure sterility, and subjected to an additional ultracentrifugation step under identical conditions (100,000× *g*, 80 min, 4 °C) for *P. g*-OMV purification. The final *P. g*-OMV pellet was resuspended in 100 μL PBS, and stored at −80 °C until further use.

The protein concentration of the *P. g*-OMV preparation was determined using a BCA protein assay kit (Cat No: PC0021, Solarbio) according to the manufacturer’s instructions, with BSA as the standard. All measurements were performed in triplicate, and the results were expressed as mean ± SD.

The total protein yield of the OMV preparation was typically 100–150 µg per liter of bacterial culture (OD_600_ ≈ 1.0). This yield was obtained from the final pellet resuspended in 100 μL PBS, resulting in a stock concentration of approximately 1000–1500 µg/mL.

### 2.2. Identification of P. g-OMVs

#### 2.2.1. Transmission Electron Microscopy (TEM)

For morphological characterization, *P. g*-OMV samples were adsorbed onto 300-mesh carbon-coated copper grids at room temperature for 5 min. After removal of excess sample with filter paper, the grids were negatively stained with 2% (*w*/*v*) aqueous uranyl acetate solution for 1 min. Excess stain was removed, and the grids were air-dried at room temperature for 3 min. Imaging was performed using a transmission electron microscope (Cat No: HT-7700, Hitachi, Tokyo, Japan) operating at an acceleration voltage of 100 kV. Images were captured randomly from multiple fields of view to ensure representativeness of *P. g*-OMV morphological characteristics.

#### 2.2.2. Nanoparticle Tracking Analysis (NTA)

Particle size distribution and concentration of *P. g*-OMV preparations were determined by nanoparticle tracking analysis in accordance with the MISEV2023 guidelines. *P. g*-OMV samples were appropriately diluted with sterile PBS to achieve a particle concentration within the optimal detection range of 1 × 10^7^–1 × 10^9^ particles/mL. Diluted samples were loaded into the sample chamber of a nanoparticle tracking analyzer (Cat No: N30E, NanoFCM, Xiamen, China). The instrument acquired and analyzed scattered light signals from individual particles to determine the size distribution, average particle size, and concentration of the *P. g*-OMVs.

#### 2.2.3. Western Blot Analysis of GRP94

*P. g*-OMV proteins were extracted using RIPA lysis buffer (Cat No: P0013B, Beyotime, Shanghai, China) supplemented with 1% (*v*/*v*) protease inhibitor cocktail (Cat No: HY-K0010, MCE, Monmouth Junction, NJ, USA). Western blot analysis was performed as described in Section 2.9 with the following modifications: 20 μg of *P. g*-OMV protein was loaded per lane. To exclude the possibility of mammalian exosome contamination, the membrane was incubated with an antibody against GRP94 (1:1000 dilution, Cat No: R381784, Rabbit, Zenbio, Chengdu, China) as a negative marker. The absence of the GRP94 signal confirmed the purity of the OMV preparation from culture-derived extracellular vesicles.

### 2.3. Cell Culture

The mouse aortic smooth muscle cell (MOVAS) (Cat No: ZQ1056, Shanghai ZQXZ Biotechnology Co., Ltd., Shanghai, China) was maintained in DMEM (Cat No: PM150210, Procell, Wuhan, China) supplemented with 10% (*v*/*v*) FBS (Cat No: 164250, Procell) and 1% (*v*/*v*) penicillin-streptomycin solution (Cat No: PB180120, Procell). Cells were incubated at 37 °C in a humidified atmosphere containing 5% CO_2_. Cells at passages 3–8 were used for all experiments.

### 2.4. Cell Viability Assay

Cell viability was assessed using the Cell Counting Kit-8 (CCK-8) assay. MOVAS cells were seeded into 96-well plates at a density of 4 × 10^3^ cells per well and allowed to adhere for 24 h. The cells were then exposed to various concentrations of *P. g*-OMVs (0, 0.5, 1, 1.5, 2, 2.5, 5, and 10 μg/mL) for an additional 24 h. Following treatment, the culture medium was replaced with 100 μL of fresh medium containing 10% (*v*/*v*) CCK-8 reagent (Cat No: C0037, Beyotime), and the plates were incubated at 37 °C for 3 h in the dark, and the optical density (OD) at 450 nm was measured. Cell viability (%) was calculated as follows: [(OD experiment − OD blank)/(OD control − OD blank)] × 100%.

### 2.5. Enzyme-Linked Immunosorbent Assay (ELISA)

MOVAS cells were seeded into 6-well plates at an appropriate density and cultured until reaching approximately 80% confluence. The cells were then treated with 2.5 μg/mL *P. g*-OMVs or an equal volume of PBS as a vehicle control for 24 h. Following incubation, cell culture supernatants were collected and centrifuged at 1000× *g* for 10 min at 4 °C to remove cellular debris. The concentrations of Col1 (Cat No: ED-24450, LunChangShuo Biotech, Xiamen, China), Col3 (Cat No: ED-22986, LunChangShuo Biotech, Xiamen, China), MMP-9 (Cat No: JL29652, Jonlnbio, Shanghai, China), MMP-2 (Cat No: JL20388, Jonlnbio) and TIMP-1 (Cat No: ED-20382, LunChangShuo Biotech) were quantified using commercially available ELISA kits according to the manufacturers’ protocols.

### 2.6. Confocal Microscopy for P. g-OMV Internalization by MOVAS Cells

*P. g*-OMVs were labeled with PKH26 red fluorescent cell linker dye (Cat No: C2071S, Beyotime) according to the manufacturer’s instructions. Briefly, OMVs were mixed with PKH26 dye and Diluent C in a 1:9 ratio, incubated for 15 min at room temperature protected from light, and then washed three times by ultracentrifugation (100,000× *g* for 20 min, 4 °C).

MOVAS cells were seeded onto sterile glass coverslips placed in 6-well plates at a density of 2.0 × 10^4^ cells per well and cultured for 24 h. Cells were then incubated with PKH26-labeled OMVs (2.5 μg/mL) or PKH26-labeled PBS for 24 h. After incubation, cells were fixed with 4% (*w*/*v*) paraformaldehyde (Cat No: P0099, Beyotime), permeabilized with 0.1% (*v*/*v*) Triton X-100 (Cat No: P0096, Beyotime) for 10 min, stained with Alexa Fluor 488-conjugated phalloidin (Cat No: C2232, Beyotime), and counterstained with DAPI (Cat No: C1002, Beyotime). Fluorescence images were acquired using a confocal laser scanning microscope (Cat No: LSM 800, Zeiss, Oberkochen, Germany).

### 2.7. Wound-Healing Assay

Wound-healing capacity was assessed using an in vitro wound-healing assay. MOVAS cells were seeded into 6-well plates at a density of 6 × 10^5^ cells per well and cultured until reaching 100% confluence. The cell monolayer was then serum-starved for 24 h. A linear scratch wound was created in the center of each well using a sterile 200 μL pipette tip. After washing away detached cells with PBS, the cultures were treated with 2.5 μg/mL *P. g*-OMVs or PBS in serum-free medium. Wound closure was monitored and photographed at 0, 9, and 18 h post-scratching using an inverted phase-contrast microscope. The percentage of wound closure was calculated using ImageJ V1.54g (National Institutes of Health, Bethesda, MD, USA).

### 2.8. Colony Formation Assay

MOVAS cells, including wild-type, CKAP4-overexpressing (OE-CKAP4), CKAP4-knockout (KO-CKAP4), and their corresponding empty vector controls (OE-NC, KO-NC), were seeded into 6-well plates at a density of 800 cells per well and cultured for 9 days, with the culture medium replaced every 3 days. Colonies containing ≥50 cells were fixed with 4% (*w*/*v*) paraformaldehyde for 10 min and stained with 0.4% (*w*/*v*) crystal violet (Cat No: Y26809, Beyotime) for 30 min at room temperature. After washing with PBS, colony numbers were counted using ImageJ software.

### 2.9. Western Blot Analysis

Total protein lysates were prepared from MOVAS cells using RIPA lysis and extraction buffer (Cat No: P0013B, Beyotime) supplemented with protease inhibitor cocktail. Protein concentrations were determined using the BCA Protein Assay Kit according to the manufacturer’s instructions. Equal amounts of protein (20–40 μg per lane) were separated by 10% sodium dodecyl sulfate-polyacrylamide gel electrophoresis (SDS-PAGE) and transferred onto 0.45 μm polyvinylidene difluoride (PVDF) membranes (Cat No: ISEQ00010, Millipore) using a wet transfer system at 200 mA for 90 min at 4 °C.

The membranes were blocked with protein-free rapid blocking buffer (Cat No: PS108, Epizyme Biotech, Shanghai, China) for 15–20 min at room temperature, primary antibodies were diluted in primary antibody dilution buffer (Cat No: PS114, Epizyme Biotech, Shanghai, China), followed by overnight incubation at 4 °C with the following primary antibodies: rabbit anti-GAPDH antibody (1:10,000 dilution, Cat No: R380626, Rabbit, Zenbio), rabbit anti-CKAP4 antibody (1:1000 dilution, Cat No: 161564, Rabbit, Zenbio), rabbit anti-MMP-2 antibody (1:1000 dilution, Cat No: 10373-2-AP, Proteintech, Wuhan, China), rabbit anti-fibronectin (FN) antibody (1:1000 dilution, Cat No: 250073, Zenbio), rabbit anti-osteopontin (OPN) antibody (1:1000 dilution, Cat No: 340690, Rabbit, Zenbio), rabbit anti-α-SMA antibody (1:7000 dilution, Cat No: R23450, Zenbio), rabbit anti-integrin alpha5 (ITGα5) antibody (1:1000 dilution, Cat No: R381532, Rabbit, Zenbio), and rabbit anti-integrin beta1 (ITGβ1) antibody (1:1000 dilution, Cat No: R24729, Rabbit, Zenbio).

After washing three times with Tris-buffered saline containing 0.1% Tween-20 (TBST), the membranes were incubated with horseradish peroxidase (HRP)-conjugated goat anti-rabbit IgG secondary antibody(1:9000 dilution, Cat No: 550109, Zenbio) for 1–2 h at room temperature. Immunoreactive bands were visualized using supersignal west pico plus chemiluminescent substrate (Cat No: 34580, Thermo Fisher Scientific, Waltham, MA, USA) and detected using a chemiluminescence imaging system. Band intensities were quantified using ImageJ software and normalized to GAPDH as the internal loading control.

### 2.10. Using the CRISPR/Cas9 System to Generate Stable Cell Lines with CKAP4 Knockout and Overexpression

#### 2.10.1. sgRNA Design

Exon sequences of the mouse Ckap4 gene (NCBI Gene ID:216197; RefSeq: NM_175451.1) were retrieved from the NCBI database. Single-guide RNAs (sgRNAs) targeting Ckap4 were designed using the ChopChop online tool (http://chopchop.cbu.uib.no/ (accessed on 2 June 2025)). Based on optimal cleavage efficiency and minimal off-target effects, the sgRNA with the highest on-target score was selected. The sgRNA target sequence is: tctgtcggacgggatccacgtgg (5′→3′). A pair of PCR primers flanking the sgRNA target site was designed for genotyping validation: forward primer gctaacgtctggattttggtcgtaggtccaatc; reverse primer cttcagagttttcacgtcctgactgcgatc.

#### 2.10.2. Lentivirus Packaging and Generation of Stable Cell Lines

Lentiviruses for Ckap4 knockout (lentiCRISPR v2-sgCkap4) and overexpression (pLV-EF1a-Ckap4-IRES-Puro) were packaged and validated by Shanghai Genechem Co., Ltd. (Shanghai, China). MOVAS cells were seeded at a density of 2 × 10^5^ cells/well in 6-well plates 24 h prior to transduction. Cells were transduced with lentiviral particles in the presence of polybrene (5 μg/mL). After 72 h, successfully transduced cells were selected with puromycin (4 μg/mL) for 7–10 days to establish the following stable cell lines: knockout empty vector control (KO-NC), Ckap4 knockout (KO-Ckap4), overexpression empty vector control (OE-NC), and Ckap4 overexpression (OE-Ckap4). Knockout and overexpression efficiencies were verified by Western blotting.

### 2.11. Statistical Analysis

Each experiment was performed using independently prepared batches of *P. g*-OMVs and freshly thawed MOVAS cells (passages 3–8) to ensure biological reproducibility. Quantitative data were first tested for normality using the Shapiro–Wilk test. Normally distributed data are presented as mean ± standard deviation (SD). Comparisons between two groups were analyzed using a two-tailed unpaired Student’s *t*-test. Multiple group comparisons were performed using one-way analysis of variance (ANOVA) followed by Dunnett’s post hoc test to compare each experimental group with the control group. Non-normally distributed data are presented as median with interquartile range and were analyzed using non-parametric tests. A *p*-value < 0.05 was considered statistically significant. All statistical analyses were conducted using GraphPad Prism 10.0 (GraphPad Software, San Diego, CA, USA).

## 3. Results

### 3.1. Isolation and Characterization of P. g-OMVs

**Figure 1:**
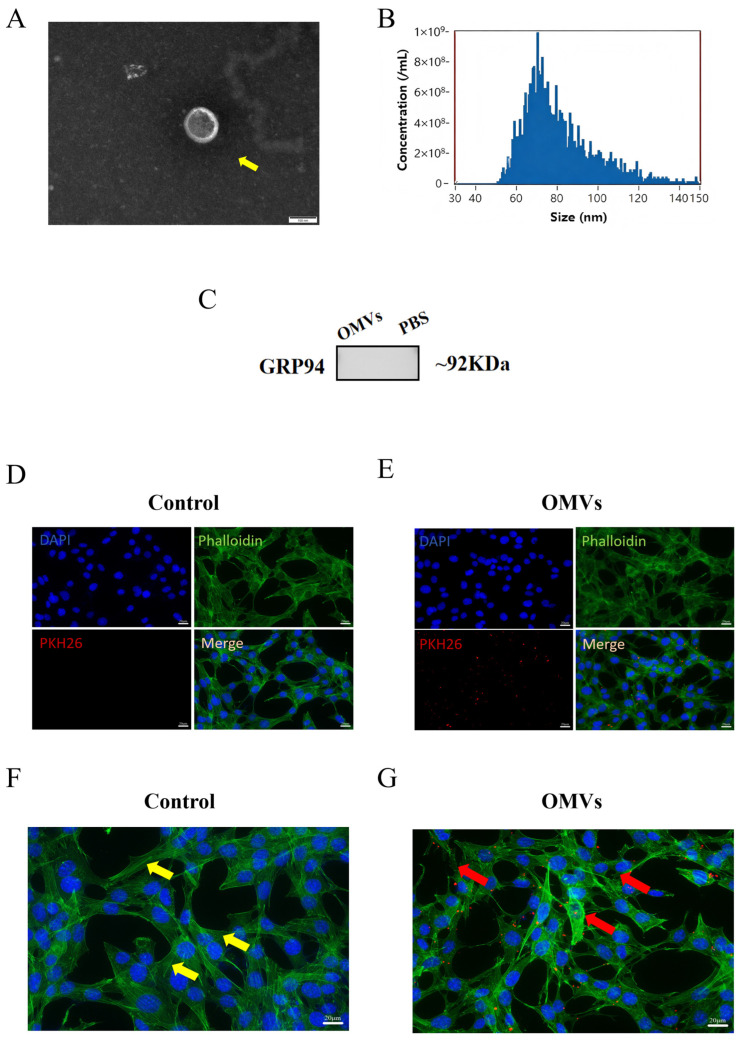
(**A**) TEM image of the sample morphology (scale bar: 100 nm); (**B**) NTA of the sample size distribution; (**C**) Western blot bands of GRP64; (**D**) Laser confocal microscopy images of contro group; (**E**) Internalization of PKH26-labeled OMVs (red) by MOVAS detected by laser confocal microscopy; nuclei were stained with DAPI (blue) (scale bar, 20 µm); (**F**) Cytoskeletal changes of control group, F-actin were stained with phalloidin (green), the yellow arrows indicate the normal cytoskeleton; (**G**) Cytoskeletal changes of OMV group, the red arrows indicate cytoskeletal disorganization and deformation. (scale bar: 20 μm).

*P. g*-OMVs were isolated from the culture supernatants of *P. gingivalis* via ultracentrifugation and were subsequently subjected to comprehensive characterization using multiple analytical approaches. TEM revealed that the isolated OMVs exhibited typical quasi-spherical vesicular structures, with yellow arrows marking the positions of the target vesicles (Figure 1A). This characteristic cup-shaped morphology was consistent with the canonical features of bacterial outer membrane vesicles. NTA was further utilized to characterize the particle size distribution of the vesicle preparation. The results revealed that the isolated OMVs had diameters ranging from 30 to 150 nm, with an average diameter of approximately 80.7 nm (Figure 1B). These size parameters were highly consistent with the previously reported size range for *P. g*-OMVs.

To further validate the purity and specificity of the isolated OMVs, Western blot analysis revealed that the negative control marker GRP94 was undetectable (Figure 1C). Collectively, these findings, including morphological observation, particle size analysis, and specific protein detection, confirmed the successful isolation of pure *P. g*-OMVs, which were utilized for the subsequent functional investigations.

### 3.2. Internalization of P. g-OMVs by MOVAS Cells and Cytoskeletal Changes

To assess whether MOVAS cells are capable of internalizing *P. g*-OMVs, isolated vesicles were labeled with the red fluorescent dye PKH26; samples subjected to the identical procedure with sterile PBS in place of OMVs served as the blank control. Following a 12 h co-incubation of MOVAS cells with either labeled *P. g*-OMVs or the PBS control, nuclei were counterstained with DAPI, and fluorescence distribution was visualized by confocal laser scanning microscopy (CLSM). In cells exposed to PKH26-labeled OMVs, distinct red fluorescence was evident, accumulating predominantly in the perinuclear region (Figure 1D). By contrast, no detectable red signal was observed within cells from the blank control incubation (Figure 1E). These observations suggested that *P. g*-OMVs could be internalized by MOVAS cells within 12 h, indicating their potential to deliver bacterial virulence factors into host cells.

F-actin constitutes the principal cytoskeletal element of VSMCs, where it not only preserves cell shape but also actively governs contraction, migration, and phenotypic modulation. Maintaining the orderly assembly of F-actin into well-defined bundles is therefore crucial for normal vascular tissue architecture and function. Staining with fluorescently conjugated phalloidin, which binds F-actin with high specificity, permits direct visualization of its subcellular distribution. CLSM imaging revealed that in untreated control MOVAS cells, F-actin organized into dense, longitudinally aligned parallel bundles that extended along the major axis of the cell, presenting a continuous and well-preserved cytoskeletal network (Figure 1F). Upon exposure to 2.5 µg/mL *P. g*-OMVs for 24 h, this structural integrity was severely compromised: the intracellular microfilament array became markedly disordered, and the characteristic parallel bundling pattern was entirely lost (Figure 1G).

### 3.3. Cell Response of Mouse Aortic Vascular Smooth Muscle Cells After Treatment with P. g-OMVs

#### 3.3.1. Effects of Different Concentrations of *P. g*-OMVs on MOVAS Cell Proliferation

To investigate the effects of *P. g*-OMVs on the proliferative activity of MOVAS cells, cultures were exposed to a graded concentration series of the vesicles, and cell viability was subsequently assessed using the CCK-8 assay. Specifically, seven concentrations of *P. g*-OMVs (0.5, 1, 1.5, 2, 2.5, 5, and 10 μg/mL) were tested over a 24 h treatment period, after which the corresponding cell survival rates were calculated and subjected to inter-group statistical analysis. Following the 24 h stimulation period, exposure to low *P. g*-OMV concentrations (0.5 and 1 μg/mL) elicited no statistically significant alteration in MOVAS cell proliferative activity when compared with the blank control group (*p* > 0.05).

**Figure 2:**
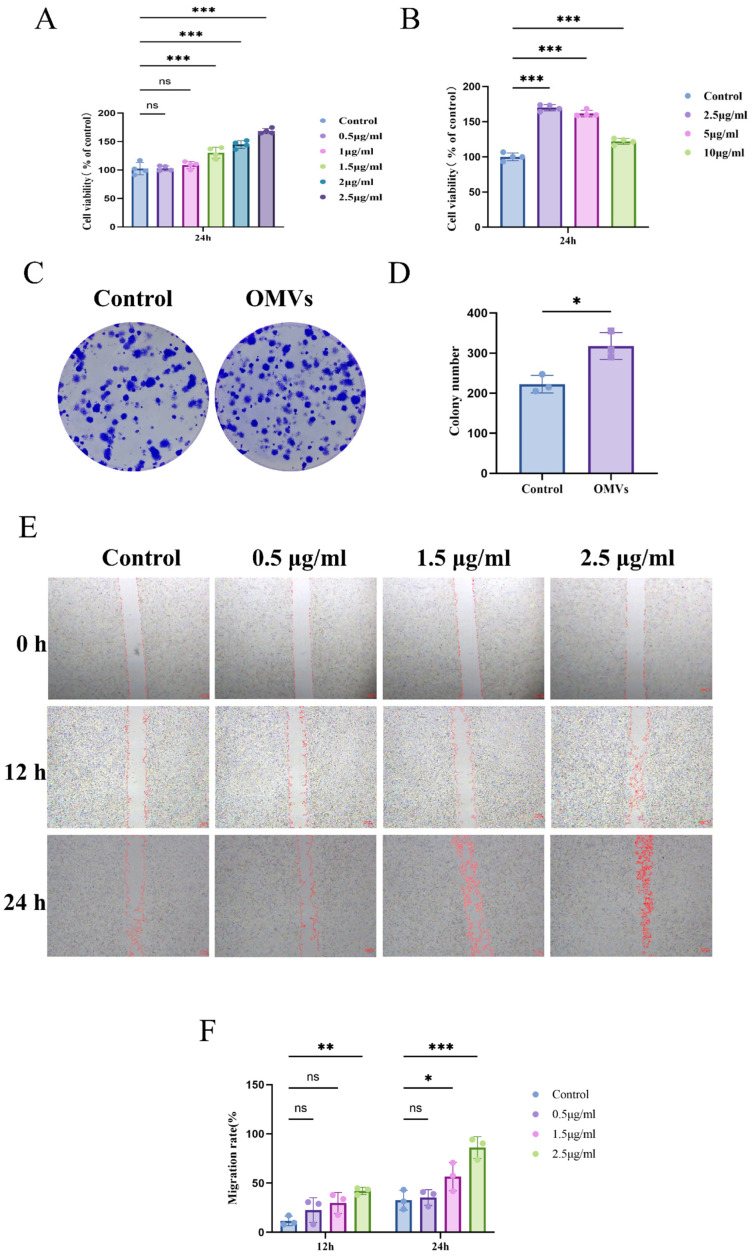

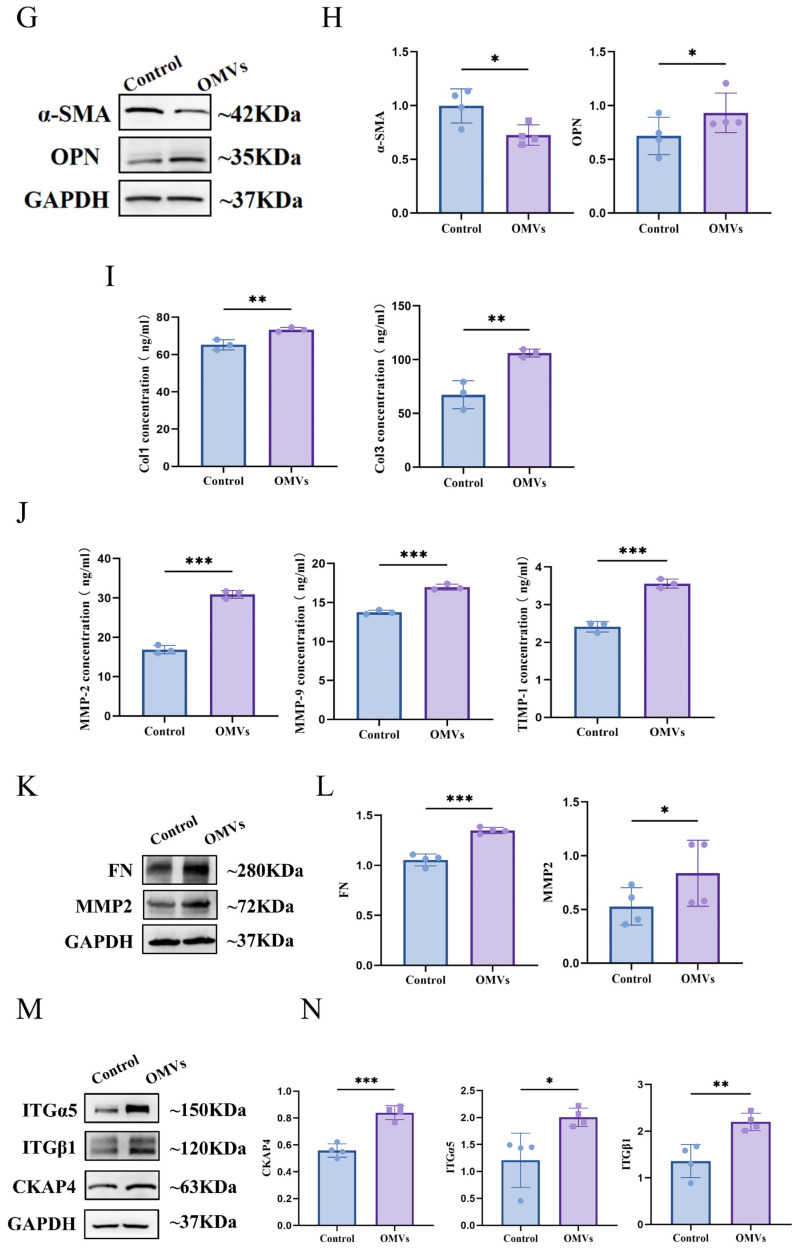
(**A**) Cell viability assessed by CCK-8 assay, low concentration range (0–2.5 μg/mL); (**B**) Cell viability assessed by CCK-8 assay, high concentration range (2.5–10 μg/mL); (**C**) Image of colony formation stained with crystal violet; (**D**) Quantitative analysis of (**C**); (**E**) Wound-healing images (scale bar, 200 µm); (**F**) Quantitative analysis of (**E**); (**G**) Western blot bands for the contractile phenotype marker α-SMA and the synthetic phenotype marker OPN; (**H**) Protein intensity values analysis of (**G**); (**I**) ELISA quantification of Col1 and Col3 secretion levels in MOVAS cells; (**J**) ELISA quantification of MMP-2, MMP-9, and TIMP-1 expression levels in MOVAS cells; (**K**) Western blot bands of FN, MMP2; (**L**) Protein intensity values analysis of (**G**); (**M**) Western blot bands of CKAP4, ITGα5, and ITGβ1; (**N**) Protein intensity values analysis of (**M**). Statistical significance is indicated as follows: * *p* < 0.05; ** *p* < 0.01; *** *p* < 0.001.

As the OMV concentration gradually increased, cell proliferation exhibited a concentration-dependent biphasic trend, with an initial increase followed by a subsequent decrease. Peak proliferative activity was recorded in the group treated with 2.5 μg/mL OMVs (Figure 2A), a value that was significantly increased relative to that of the control condition (*p* < 0.001). Upon further elevation of the concentration to 5 μg/mL and 10 μg/mL, cell proliferation exhibited a gradual diminishment in comparison with the 2.5 μg/mL group (Figure 2B), revealing a clear downward trend. The results showed that the proliferation of MOVAS cells stimulated by *P. g*-OMVs showed a concentration-dependent response: In a specific concentration range, OMV treatment can promote cell growth; when the concentration exceeds the optimal threshold, this proliferative effect will be weakened.

#### 3.3.2. Effects of *P. g*-OMVs on the Colony Formation Ability of MOVAS Cells

To further validate the conclusion from the prior CCK-8 assay that *P. g*-OMVs promote the proliferation of MOVAS cells, a colony formation assay was performed using the optimal pro-proliferative concentration of *P. g*-OMVs (2.5 μg/mL), as identified in the previous experiment.

Compared with the blank control condition, the *P. g*-OMV-stimulated group displayed a marked elevation in the number of discernible cell colonies (Figure 2C). Quantitative analysis revealed that the colony formation capacity was significantly enhanced, with a statistically significant difference between the two groups (*p* < 0.05) (Figure 2D).

#### 3.3.3. The Effect of *P. g*-OMVs on the Wound-Healing Ability in MOVAS Cells

In order to explore the effect of *P. g*-OMVs on wound closure, the wound-healing experiment was used in this study to observe the wound closure at 0 h, 9 h, and 18 h after treatment with different concentrations of *P. g*-OMVs. After 9 and 18 h of incubation, groups exposed to 0.5 and 1.5 μg/mL OMVs displayed a modest reduction in scratch area relative to the blank control (Figure 2E); nevertheless, these differences failed to reach the threshold of statistical significance (*p* > 0.05) (Figure 2F).

In contrast, treatment with *P. g*-OMVs at a concentration of 2.5 μg/mL elicited a pronounced diminution in scratch area at the 24 h time point, with the observed enhancement in wound closure differing significantly from that of the control group (*p* < 0.001). It should be noted that the wound-healing assay cannot clearly distinguish the proportion of cell migration and cell proliferation in wound closure, so the effect observed in this study is regarded as a comprehensive biological response mediated by both.

#### 3.3.4. Effects of *P. g*-OMVs on Phenotypic Transformation of MOVAS Cells

Western blot analysis was performed to detect changes in the expression of phenotypic marker proteins in MOVAS cells following stimulation. Following a 24 h exposure to 2.5 μg/mL *P. g*-OMVs, a marked downregulation of the contractile phenotype marker α-SMA was observed, accompanied by a concurrent upregulation of the synthetic phenotype marker OPN (Figure 2G); both alterations differed significantly from the corresponding levels in the control group (*p* < 0.05) (Figure 2H). There is a correlation between *P. g*-OMV stimulation and the phenotypic shift of MOVAS cells from contractile to synthetic phenotype.

#### 3.3.5. Effects of *P. g*-OMVs on ECM Deposition and Remodeling in MOVAS Cells

To further investigate the regulatory effects of 2.5 μg/mL *P. g*-OMVs on ECM synthesis and remodeling in MOVAS cells, ELISA and Western blotting were used to quantify the levels of relevant marker proteins. Quantification by ELISA demonstrated that a 24 h exposure to 2.5 μg/mL *P. g*-OMVs led to a significant elevation in the secreted levels of the ECM structural proteins Col1 and Col3 (*p* < 0.01) (Figure 2I). A parallel upregulation was evident for the ECM remodeling proteases MMP-2 and MMP-9, as well as their endogenous inhibitor TIMP-1 (*p* < 0.001) (Figure 2J).

Western blot analysis provided further corroboration that 2.5 μg/mL *P. g*-OMVs effectively enhanced MMP-2 protein expression and concurrently augmented the secretion of the ECM component FN (Figure 2K), with the difference achieving statistical significance (*p* < 0.05, *p* < 0.001) (Figure 2L). Taken together, these data revealed that after 2.5 μg/mL *P. g*-OMV treatment, extracellular matrix protein synthesis increased and matrix metalloproteinase activity increased, thereby promoting both the deposition of matrix constituents (including collagen and fibronectin) and the initiation of ECM remodeling.

#### 3.3.6. Effects of *P. g*-OMVs on CKAP4, ITGα5, and ITGβ1 Protein Levels in MOVAS Cells

Western blot analysis was performed to measure the protein expression levels of CKAP4, ITGα5, and ITGβ1 in MOVAS cells following 24 h of stimulation with 2.5 μg/mL *P. g*-OMVs (Figure 2M). The results indicated that compared with the control group, the protein expression levels of CKAP4, ITGα5, and ITGβ1 were significantly upregulated in the *P. g*-OMV-treated group (*p* < 0.05, *p* < 0.01, *p* < 0.001) (Figure 2N).

### 3.4. The Role of CKAP4 in the Functional Changes of MOVAS Cells Caused by P. g-OMVs

#### 3.4.1. Validation of Lentiviral Overexpression and Knockout Efficiency of CKAP4 in MOVAS Cells

**Figure 3:**
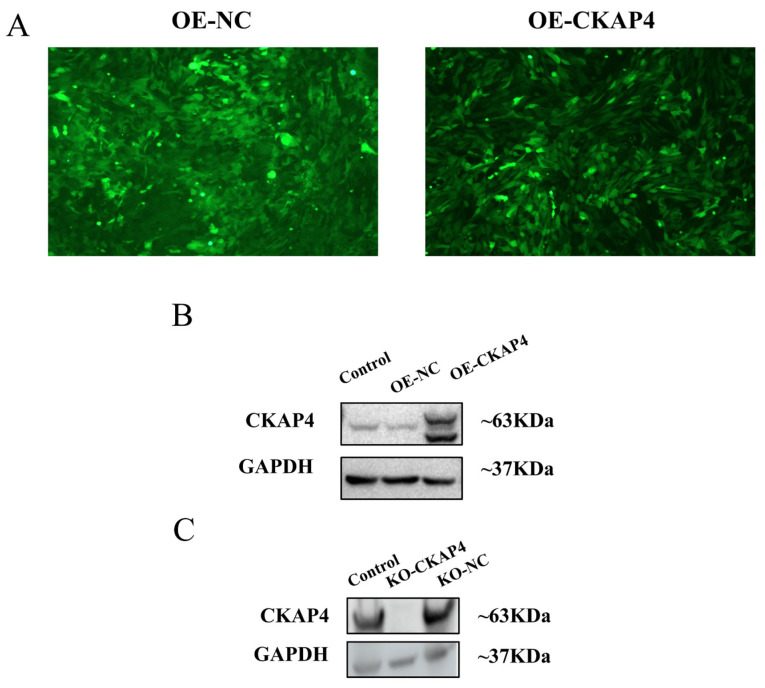
(**A**) Fluorescence images of the OE-NC group and OE-CKAP4 group; The KO group lacks a GFP tag, and no fluorescence images were acquired. (**B**) Validation of CKAP4 protein levels after overexpression. (**C**) Validation of CKAP4 protein levels after knockout.

72 h after lentiviral transfection, the majority of MOVAS cells in the CKAP4 overexpression group and CKAP4 overexpression-negative control group, both carrying a GFP reporter, displayed clear green fluorescence under fluorescence microscopy (Figure 3A). In contrast, the CKAP4 knockout group was established with a CRISPR/Cas9 lentiviral vector containing a puromycin resistance gene but no GFP tag. Accordingly, no images were collected for the knockout group, and the knockout efficiency was verified solely by Western blot analysis.

In order to evaluate whether both CKAP4 overexpression and knockout cell models were successfully established, MOVAS cells from each lentivirally transduced population were harvested and subjected to total protein extraction for Western blot analysis. In the overexpression arm, CKAP4 protein levels in the OE-NC group remained comparable to those of the control condition (*p* > 0.05), whereas a robust and significant elevation in CKAP4 expression was observed in the OE-CKAP4 group (*p* < 0.001) (Figure 3B). Within the knockout experiment, CKAP4 expression in the KO-NC group did not deviate appreciably from control levels (*p* > 0.05); by contrast, the KO-CKAP4 group exhibited a pronounced reduction in CKAP4 protein abundance relative to the control (*p* < 0.001). This finding verifies that both the CKAP4 overexpression and knockout cell models were successfully established at the protein level (Figure 3C).

#### 3.4.2. Effects of CKAP4 Overexpression on Colony Formation Ability of MOVAS Cells

**Figure 4:**
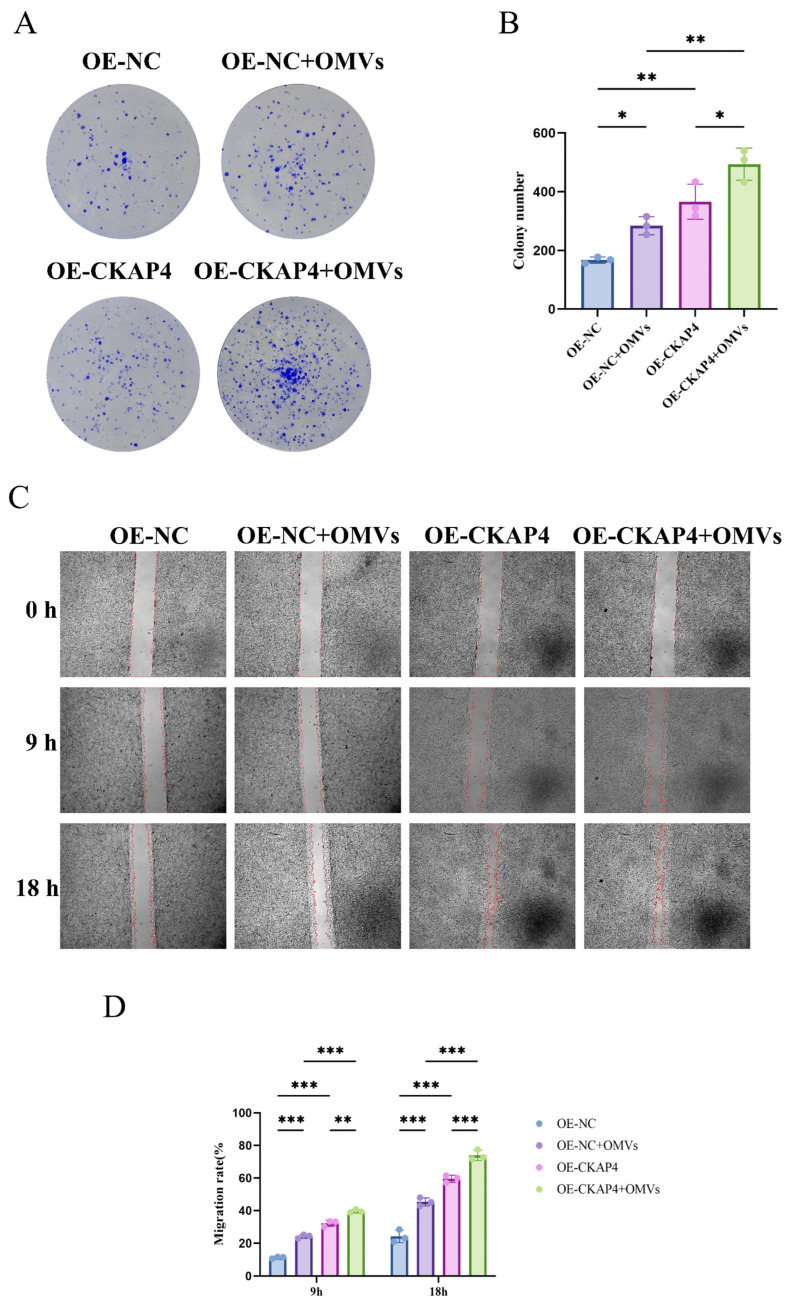

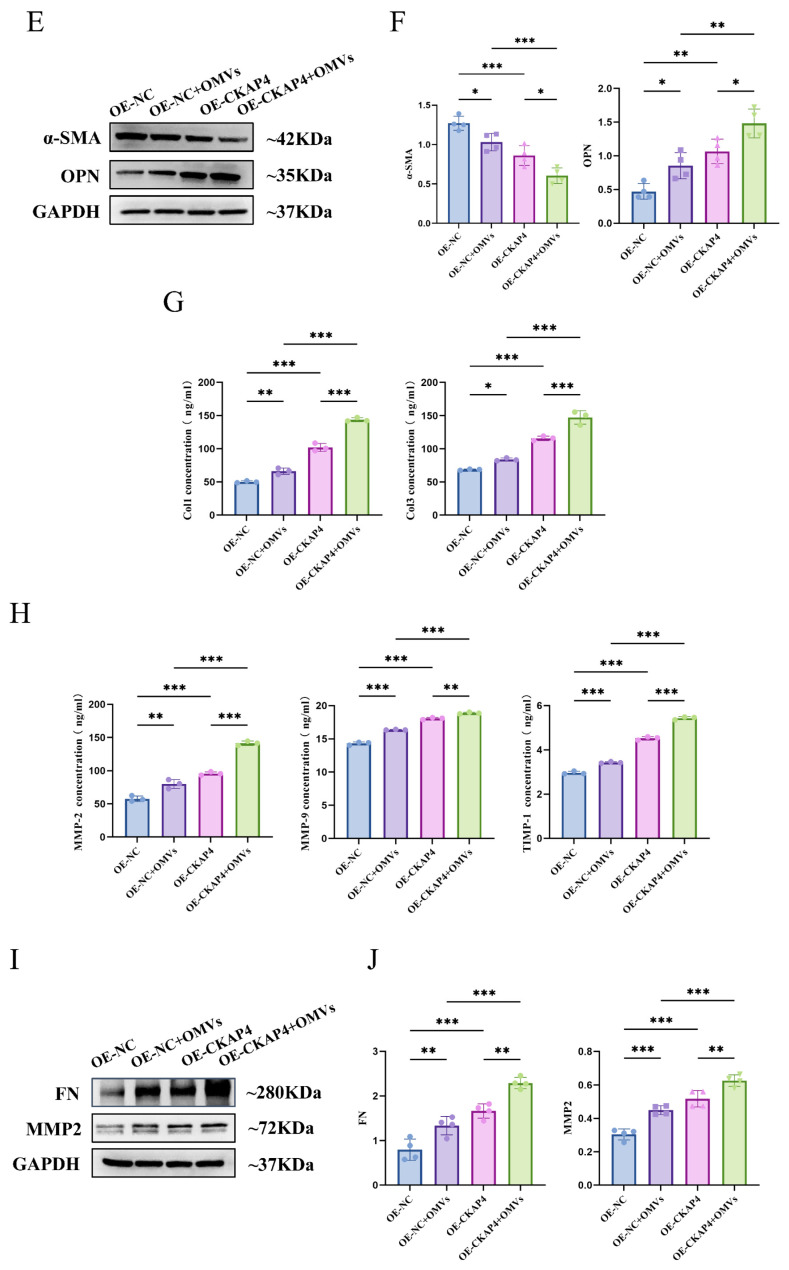

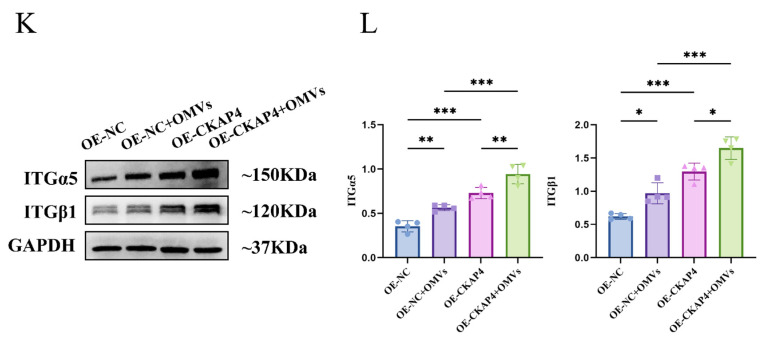
(**A**) Image of colony formation stained with crystal violet; (**B**) Quantitative analysis of (**B**); (**C**) Wound-healing assay images (scale bar, 200 µm); (**D**) Quantitative analysis of (**C**); (**E**) Western blot bands for the contractile phenotype marker α-SMA and the synthetic phenotype marker OPN; (**F**) Protein intensity values analysis of (**E**); (**G**) ELISA quantification of Col1 and Col3 secretion levels in MOVAS cells; (**H**) ELISA quantification of MMP-2, MMP-9, and TIMP-1 expression levels in MOVAS cells; (**I**) Western blot bands of FN, MMP2; (**J**) Protein intensity values analysis of (**I**); (**K**) Western blot bands of ITGα5, and ITGβ1; (**L**) Protein intensity values analysis of (**K**). Statistical significance is indicated as follows: * *p* < 0.05; ** *p* < 0.01; *** *p* < 0.001.

To explore whether CKAP4 modulates the proliferative behavior of MOVAS cells, we carried out colony formation assays to evaluate changes in cell clonogenic ability under CKAP4 overexpression, as well as following combined intervention with *P. g*-OMVs. Compared with the OE-NC control group, cells with CKAP4 overexpression exhibited a prominent increase in colony numbers (*p* < 0.01), implying that upregulated CKAP4 expression correlates with the clonogenic growth of MOVAS cells. After incubation with 2.5 μg/mL *P. g*-OMVs, the OE-NC+OMV group displayed evidently elevated colony formation relative to the basal OE-NC group (*p* < 0.05) (Figure 4A), further validating the pro-proliferative effect of *P. g*-OMVs on vascular smooth muscle cells. Moreover, the OE-CKAP4+OMV group showed the strongest clonogenic ability among all experimental groups, with significantly increased colony counts (*p* < 0.01) (Figure 4B). This pattern reveals a synergistic pro-proliferative action between CKAP4 upregulation and *P. g*-OMV exposure.

These results collectively indicate that CKAP4 overexpression potentiates the promotional effect of *P. g*-OMVs on MOVAS colony formation, suggesting that CKAP4 plays an important regulatory role in OMV-associated vascular smooth muscle cell proliferation.

#### 3.4.3. Effects of CKAP4 Overexpression on Wound-Healing in MOVAS Cells

A wound-healing assay was used to explore the effect of CKAP4 overexpression on wound closure of MOVAS cells (which integrates both migration and proliferation), and the degree of wound closure was quantitatively detected at 9 and 18 h after scratch treatment. With the extension of culture duration, all groups displayed gradual wound closure, and the wound closure degree at 18 h was markedly higher than that at 9 h across all experimental settings (Figure 4C). At both time points, cells overexpressing CKAP4 showed a remarkably faster wound closure rate than the OE-NC control group (*p* < 0.001), indicating that CKAP4 upregulation inherently promotes MOVAS cell wound closure. After further stimulation with 2.5 μg/mL *P*. *g*-OMVs, the cell wound closure effect was significantly enhanced. Notably, the OE-CKAP4+OMV group achieved an approximately 80% wound closure rate at 18 h, which was significantly superior to all other treatment groups (*p* < 0.001) (Figure 4D). Collectively, these data indicate that elevated CKAP4 expression acts synergistically with *P. g*-OMV exposure to robustly facilitate MOVAS wound closure.

#### 3.4.4. Effects of CKAP4 Overexpression on Phenotypic Transformation of MOVAS Cells

We next explored whether CKAP4 upregulation modulates the phenotypic switching of MOVAS cells triggered by *P. g*-OMVs. The protein abundances of the contractile marker α-SMA and the synthetic marker OPN were determined via Western blot. Compared with OE-NC cells, the OE-CKAP4 group showed a remarkable downregulation of α-SMA (*p* < 0.001) and a notable increase in OPN expression (*p* < 0.01) (Figure 4E). After 24 h stimulation with 2.5 μg/mL *P. g*-OMVs, both OE-NC and OE-CKAP4 cells displayed a consistent trend: α-SMA was distinctly decreased while OPN was significantly upregulated relative to their corresponding untreated controls (*p* < 0.05) (Figure 4F).

#### 3.4.5. Effects of CKAP4 Overexpression on ECM Deposition and Remodeling in MOVAS Cells

We further explored the influence of CKAP4 upregulation on ECM deposition and remodeling triggered by *P. g*-OMVs in MOVAS cells, and quantified the associated protein levels using ELISA combined with Western blot. ELISA results showed that compared with the OE-NC group, the secretion levels of Col1 and Col3 in the ECM of the OE-CKAP4 group were significantly elevated (*p* < 0.001). Following stimulation with 2.5 μg/mL *P. g*-OMVs, Col1 and Col3 secretion further increased (Figure 4G). Compared with the OE-NC group, the levels of MMP-2, MMP-9, and TIMP-1 in the OE-NC+OMV group were significantly upregulated (*p* < 0.001). Relative to the OE-NC+OMV condition, the OE-CKAP4+OMV group exhibited further enhancement in the levels of MMP-2, MMP-9, and TIMP-1 (*p* < 0.001) (Figure 4H).

Western blot analysis further validated these trends (Figure 4I). Quantitative analysis showed that compared with the OE-NC group, the levels of FN and MMP-2 in the OE-CKAP4 group were significantly increased (Figure 4J) (*p* < 0.001). Following *P. g*-OMV stimulation, FN and MMP-2 expression in both the OE-NC+OMV and OE-CKAP4+OMV groups was markedly elevated relative to their respective unstimulated controls, observations that align closely with the ELISA findings.

In summary, these data suggest that overexpression of CKAP4 significantly enhances the expression of Col1, Col3, MMP-2, MMP-9, and TIMP-1 in MOVAS.

#### 3.4.6. Effects of CKAP4 Overexpression on ITGα5 and ITGβ1 Protein Levels in MOVAS Cells

To uncover the potential mechanism underlying CKAP4-mediated cellular responses to *P. g*-OMVs, we examined the protein levels of CKAP4-interacting molecules ITGα5 and ITGβ1 by Western blot. Compared with the OE-NC control, stable CKAP4 overexpression markedly elevated the protein abundance of both ITGα5 and ITGβ1 (*p* < 0.001) (Figure 4K,L). After *P. g*-OMV intervention, ITGα5 and ITGβ1 expression were further upregulated in both OE-NC+OMV and OE-CKAP4+OMV groups (*p* < 0.01). Collectively, these data indicate that CKAP4 upregulation intrinsically increases ITGα5/ITGβ1 expression, and *P. g*-OMV treatment exerts an additive promoting effect on their expression.

#### 3.4.7. Effects of CKAP4 Knockout on Colony Formation Ability of MOVAS Cells

**Figure 5:**
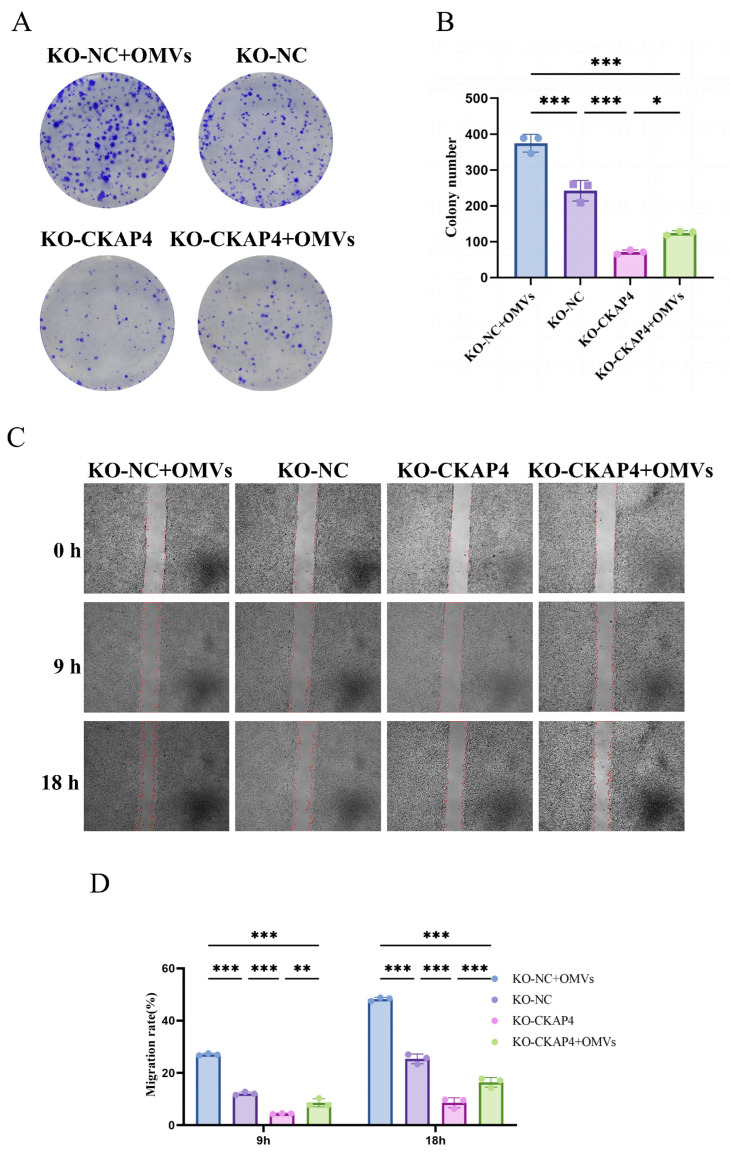

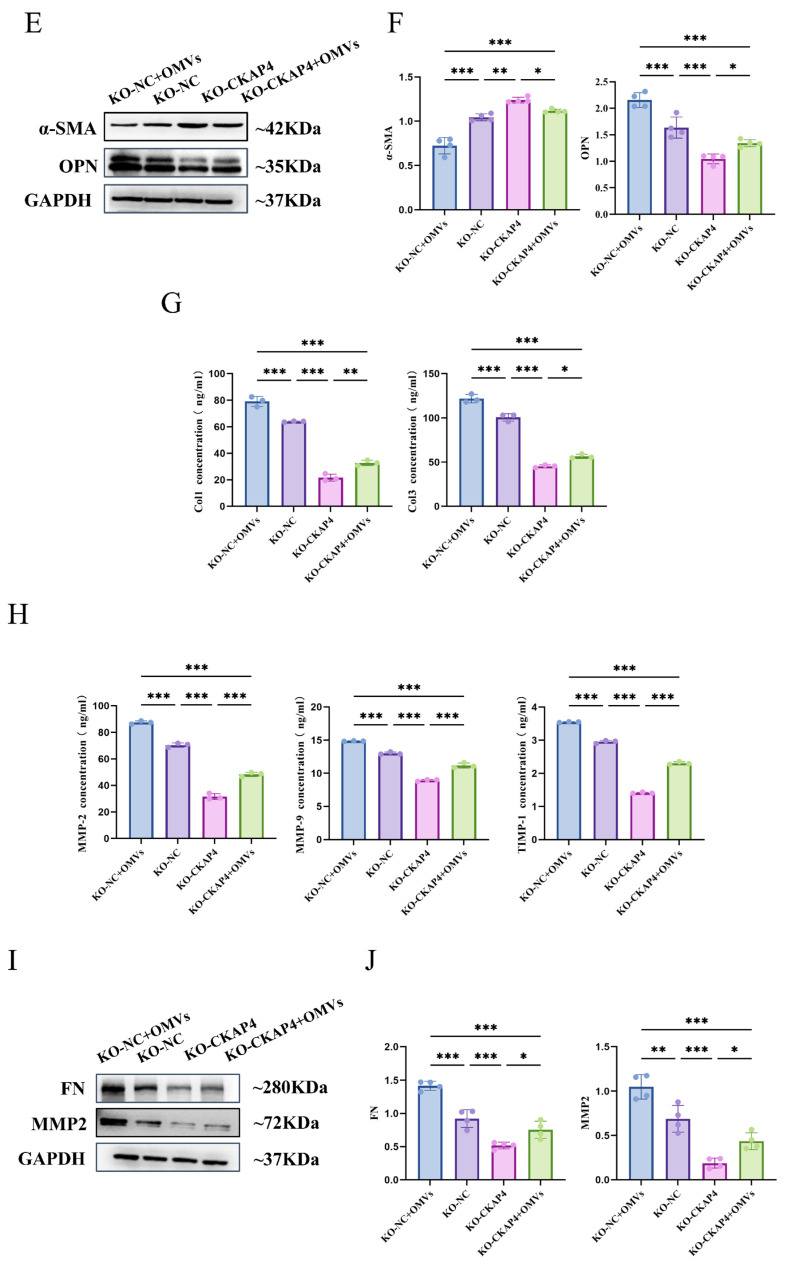

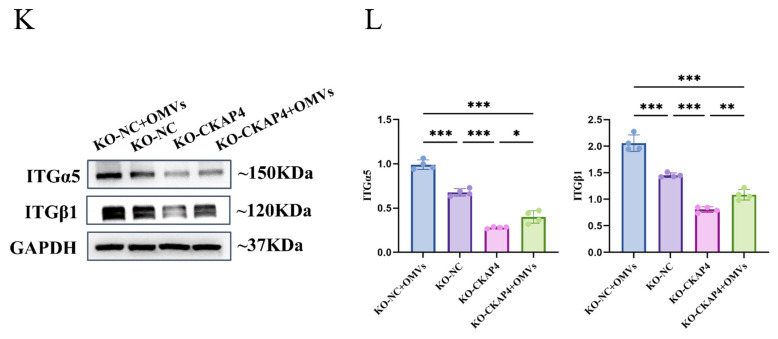
(**A**) Colony formation visualized by crystal violet stainingt; (**B**) Quantification of colony formation shown in (A); (**C**) Representative images from wound-healing assays (scale bar, 200 µm); (**D**) Quantification of wound closure presented in (C); (**E**) Western blot analysis of the contractile marker α-SMA and the synthetic marker OPN; (**F**) Protein intensity values analysis of (**E**); (**G**) Secretion levels of Col1 and Col3 measured by ELISA in MOVAS cells; (**H**) Expression levels of MMP-2, MMP-9 and TIMP-1 determined by ELISA in MOVAS cells; (**I**) Western blot bands of FN, MMP2; (**J**) Quantitative analysis of band intensities for FN and MMP2 (I); (**K**) Western blot bands of ITGα5, and ITGβ1; (**L**) Protein intensity values analysis of (**K**). Statistical significance is indicated as follows: * *p* < 0.05; ** *p* < 0.01; *** *p* < 0.001.

To investigate the effect of CKAP4 knockout on *P. g*-OMVs-induced proliferation of MOVAS cells, a colony formation assay was used to detect the colony formation capacity of cells in each group. A marked reduction in the number of colonies was evident in the KO-CKAP4 group when compared with the KO-NC control (*p* < 0.001) (Figure 5A), demonstrating that depletion of CKAP4 impairs the proliferative potential of MOVAS cells. Upon stimulation with 2.5 μg/mL *P. g*-OMVs, colony counts in both the KO-NC+OMV and KO-CKAP4+OMV groups rose significantly above those of their respective untreated counterparts; notably, the colony number in the KO-CKAP4+OMV group remained substantially lower than that recorded for the KO-NC+OMV condition (*p* < 0.001) (Figure 5B).

It was found that the absence of CKAP4 can partially attenuate the enhanced proliferation effect of MOVAS cells induced by *P. g*-OMVs.

#### 3.4.8. Effects of CKAP4 Knockout on Wound-Healing in MOVAS Cells

To investigate the role of CKAP4 in *P. g*-OMV-regulated wound closure of MOVAS cells, a wound-healing assay was used to detect the effect of CKAP4 knockout combined with *P. g*-OMV treatment on wound-healing. The results show that as the incubation time increased, the healing capacity of cells in all groups increased significantly. At the 9 and 18 h time points, the KO-CKAP4 group exhibited significantly lower wound closure rates than the KO-NC group (*p* < 0.001).

After treatment with 2.5 μg/mL *P. g*-OMVs, the wound closure rates of all groups were elevated (Figure 5C). Notably, although the healing rate of the KO-CKAP4+OMV group was increased compared with the KO-CKAP4 group, it remained significantly lower than that of the KO-NC+OMV group (*p* < 0.001) (Figure 5D).

Knockout of CKAP4 significantly inhibited the wound closure ability of MOVAS cells and partially attenuated the *P. g*-OMV-induced enhancement of wound-healing, suggesting that CKAP4 is a key target through which *P. g*-OMVs regulate MOVAS cell migration and proliferation.

#### 3.4.9. Effects of CKAP4 Knockout on Phenotypic Transformation of MOVAS Cells

To investigate the effects of CKAP4 knockout on *P. g*-OMVs-induced phenotypic transformation of MOVAS cells, Western blot was used to measure the levels of the contractile phenotype marker α-SMA and the synthetic phenotype marker OPN.

As depicted in Figure 5E, α-SMA expression in the KO-CKAP4 group was markedly elevated relative to the KO-NC control (*p* < 0.01), whereas OPN levels were correspondingly suppressed to a significant degree (*p* < 0.001). Exposure to 2.5 μg/mL *P. g*-OMVs resulted in a pronounced reduction of α-SMA and a concurrent upregulation of OPN in the KO-NC+OMV group compared with the untreated KO-NC condition (*p* < 0.001) (Figure 5F), providing further corroboration that *P. g*-OMVs drive the phenotypic conversion of MOVAS cells. In contrast, the KO-CKAP4+OMV group exhibited elevated α-SMA expression and diminished OPN levels when compared with the KO-NC+OMV cohort (*p* < 0.001).

CKAP4 deficiency partially reversed the *P. g*-OMV-induced phenotypic transformation of MOVAS cells toward the synthetic phenotype, further supporting that CKAP4 plays a key role in this process.

#### 3.4.10. Effects of CKAP4 Knockout on ECM Deposition and Remodeling in MOVAS Cells

To investigate the effects of CKAP4 knockout on *P. g*-OMVs-induced ECM deposition and remodeling in MOVAS cells, ELISA was used to quantify the levels of relevant proteins. In the KO-CKAP4 group, Col1 and Col3 levels were markedly diminished (*p* < 0.001) (Figure 5G).

Following stimulation with 2.5 μg/mL *P. g*-OMVs, the levels of Col1 and Col3 in the KO-CKAP4+OMV group were lower, whereas those in the KO-NC+OMV group were highest (*p* < 0.001). Meanwhile, compared with the KO-NC group, the levels of MMP-2, MMP-9, and TIMP-1 in the KO-CKAP4 group were significantly decreased (*p* < 0.001). Upon *P. g*-OMV stimulation, the levels of these three proteins in the KO-CKAP4+OMV group also remained substantially lower than those measured in the KO-NC+OMV group (*p* < 0.001) (Figure 5H).

Western blot results further validated these trends. Compared with the KO-NC group, the levels of FN and MMP-2 in the KO-CKAP4 group were significantly reduced (*p* < 0.001) (Figure 5I). After 2.5 μg/mL *P. g*-OMVs were stimulated, FN and MMP-2 expression in both the KO-NC+OMV and KO-CKAP4+OMV groups was significantly elevated compared with the KO-NC and KO-CKAP4 groups (Figure 5J). Nevertheless, the expression levels of FN and MMP-2 in the KO-CKAP4+OMV group remained significantly lower than those detected in the KO-NC+OMV cohort.

It was found that the absence of CKAP4 significantly inhibited the expression of Col1, Col3, MMP-2, MMP-9, TIMP-1, and FN, corroborating the overexpression results and indicating that CKAP4 is a key positive regulator of ECM remodeling. With 24 h of *P. g*-OMV stimulation, MOVAS cells with CKAP4 knockout partially restored the expression of these proteins but did not reach the levels observed in the KO-NC group, suggesting that the regulatory effect of *P. g*-OMVs on MOVAS cell ECM is partially dependent on CKAP4.

#### 3.4.11. Effects of CKAP4 Knockout on ITGα5 and ITGβ1 Protein Levels in MOVAS Cells

To investigate the effects of CKAP4 knockout on the levels of ITGα5 and ITGβ1 in MOVAS cells, Western blot was used to measure the levels of these proteins in each group.

Collectively, these findings suggest that compared with the KO-NC group, the levels of both ITGα5 and ITGβ1 in the KO-CKAP4 group were significantly decreased (Figure 5K) (*p* < 0.001). Exposure to 2.5 μg/mL *P. g*-OMVs elicited a significant elevation in ITGα5 and ITGβ1 levels in the KO-NC+OMV group compared with the untreated KO-NC condition (*p* < 0.001) (Figure 5L). In the KO-CKAP4+OMV group, although the levels of ITGα5 and ITGβ1 were increased compared with the KO-CKAP4 group (*p* < 0.05 for ITGα5; *p* < 0.01 for ITGβ1), they remained significantly lower than those in the KO-NC+OMV group (*p* < 0.001).

Statistical analysis revealed that loss of CKAP4 significantly downregulated the levels of ITGα5 and ITGβ1 and partially attenuated the *P. g*-OMV-induced upregulation of these proteins.

## 4. Discussion

Numerous epidemiological studies and basic experimental investigations have confirmed a close association between chronic periodontitis and atherosclerosis [27,28,29]. However, clinical evidence has shown that antibiotic therapy fails to markedly reduce the risk of cardiovascular events in patients with both periodontitis and cardiovascular disease. Furthermore, it is difficult to detect viable *P. gingivalis* in atherosclerotic plaques. These observations cast doubt on the conventional notion that *P. gingivalis* can directly invade the vascular wall and drive the progression of atherosclerosis.

Compared with whole bacteria, OMVs are smaller in size, have greater tissue penetrance, and enhanced evasion of immune recognition. These properties enable OMVs to function effectively as long-range vehicles for the systemic dissemination of periodontal virulence factors [30,31]; accordingly, they are regarded as a crucial long-range pathogenic mediator linking local periodontal infection to systemic vascular lesions.

Previous studies have primarily focused on the direct damaging effects of *P. g*-OMVs on the vascular endothelium. However, phenotypic switching of VSMCs, along with their abnormal proliferation and migration, as well as imbalanced ECM metabolism, are equally critical. These processes govern plaque growth, structural stability, and long-term patient prognosis [32,33]. As a transmembrane protein, CKAP4 has gradually attracted attention in recent years for the regulation of cell function and inflammatory signal transduction. However, whether it is involved in mediating the functional changes in VSMCs induced by periodontal pathogen OMVs has not been elucidated.

Earlier investigations have demonstrated that *P. gingivalis* can invade human coronary VSMCs and that VSMCs are more susceptible to *P. gingivalis* virulence factors than vascular endothelial cells [34]. Our study suggested that after MOVAS cells internalize *P. g*-OMVs, the cellular cytoskeleton becomes disordered. Cytoskeletal homeostasis serves as the structural basis for regulating VSMC proliferation, migration, and phenotypic switching [35,36]. However, whether this effect is specifically mediated by the OMVs themselves or attributable to the LPS or other virulence factors they carry remains to be further confirmed.

CCK-8 assays revealed that *P. g*-OMVs exerted a clear concentration-dependent biphasic effect on MOVAS cell proliferation. At low concentrations (0.5–1 μg/mL), OMVs exhibited no significant effect on cell proliferation; the proliferative effect peaked at 2.5 μg/mL, whereas cell proliferation gradually declined at higher concentrations (5–10 μg/mL). These findings suggest that excessive OMV concentrations may exert cytotoxicity, thereby inhibiting normal cellular activity, and indicate a complex dose-dependent relationship in OMV-mediated regulation of VSMC function. Such a biphasic effect—stimulation at low concentrations and inhibition at high concentrations—has also been reported for OMVs derived from other pathogens [37]. The high-dose inhibition may reflect either cumulative cytotoxicity resulting from concentrated virulence factors, or the activation of intrinsic negative-feedback mechanisms—such as the p53/p21 axis [38] or the miR-146a/KLF4 loop [39], that restrain uncontrolled proliferation.

Colony formation and wound-healing assays further confirmed that 2.5 μg/mL *P. g*-OMVs significantly enhanced the colony-forming ability and wound closure capacity of MOVAS cells, a finding with important pathophysiological implications for early atherosclerosis. Abnormal migration of VSMCs from the media to the intima and their excessive proliferation are critical steps in atherosclerotic lesion development [40]. As mentioned in the Section 3, the outcome of the wound-healing assay reflects the combined effects of cell migration and proliferation. In the present study, although serum starvation treatment was applied to minimize cell proliferation, a Transwell migration assay, which is not affected by proliferation, was still required to further confirm that the observed effect was primarily mediated by cell migration.

Western blot analysis revealed that *P. g*-OMVs significantly downregulated the contractile marker α-SMA and upregulated the synthetic marker OPN in MOVAS cells, confirming that OMV treatment induces phenotypic switching of VSMCs from a contractile to a synthetic state. However, these two markers alone do not fully capture the entire spectrum of VSMC phenotypic changes. Future studies that examine additional contractile markers such as SM22α, calponin, and synthetic markers like vimentin will provide a more comprehensive characterization of *P. g*-OMVs-induced phenotypic switching.

After the intervention of OMVs, Col1, Col3, FN, MMP-2, MMP-9, and TIMP-1 were up-regulated simultaneously, suggesting that the ECM was in a state of high turnover remodeling rather than simple matrix deposition or degradation. From a pathophysiological perspective, this aberrant remodeling directly influences plaque progression. Excessive deposition of Col1, Col3, and FN by synthetic VSMCs drives intimal thickening, increases vascular wall stiffness, narrows the arterial lumen, and elevates circumferential mechanical stress within the vessel wall. Concurrently, elevated MMP-2 and MMP-9 activity—partially counterbalanced by TIMP-1—can degrade collagen cross-links within the fibrous cap, rendering advanced plaques susceptible to rupture and precipitating acute cardiovascular events [41]. Consequently, these observed changes may act as a double-edged sword: early ECM synthesis may facilitate reparative fibrous cap formation, whereas sustained, dysregulated remodeling under persistent OMV exposure ultimately compromises plaque stability.

Our finding that *P. g*-OMVs concurrently upregulate ECM synthesis and proteolytic remodeling aligns with the view of vascular ECM as a dynamically regulated microenvironment that actively shapes VSMCs’ fate [42]. Following OMV-induced phenotypic switching, alterations in ECM composition and stiffening may activate integrin-mediated mechanosignaling pathways such as ITGα5β1, thereby reinforcing the synthetic phenotype and sustaining migratory activity [43,44]. These observations raise the possibility of a positive feedback loop, wherein phenotypic switching may promote ECM deposition and elevated matrix stiffness, which in turn further facilitates cellular phenotypic switching, proliferation, and migration through this self-perpetuating mechanism [45,46,47].

Phillips used a complete *P. gingivalis* culture system to confirm that the IX-type secretion system of this bacterium can induce dysfunction of VSMCs [48]. Our work indicates that treatment with *P. g*-OMVs alone is associated with phenotypic transformation of VSMCs, accelerates wound-healing, and triggers ECM remodeling. Consistent with the findings of the present study, Guo demonstrated that *P. g*-OMVs could activate the ECM-receptor interaction signaling pathway through the zebrafish model [30].

In addition, our study provides preliminary evidence for a potential association between CKAP4 upregulation and *P. g*-OMV-related VSMC dysfunction. CKAP4 participates in regulating cell proliferation and migration [49]. Previous cardiovascular research has suggested that CKAP4 may influence the balance between proliferation and apoptosis in VSMCs [24]. In our experiments, treatment with *P. g*-OMVs was associated with concurrent upregulation of CKAP4 and integrin α5β1 in MOVAS cells. Overexpression of CKAP4 appeared to synergize with OMVs in promoting VSMC proliferation, wound-healing, phenotypic switching, and ECM remodeling, whereas CKAP4 knockout tended to attenuate these changes and reduce ITGα5β1 expression.

ITGα5β1 is an important integrin in VSMCs that senses mechanical signals from the ECM and plays a significant role in regulating cell adhesion, proliferation, and migration [50]. Previous studies have shown that CKAP4 can influence cell migration by regulating integrin recycling [25]. Our findings are consistent with this notion, indicating that stimulation with *P. g*-OMVs is accompanied by upregulation of CKAP4 and integrin α5β1, suggesting that these molecules may be involved in regulating the sensitivity of VSMCs to ECM mechanical signals. When ECM stiffness increases owing to collagen deposition and cross-linking, ITGα5β1 perceives this change and subsequently activates the downstream FAK/Src signaling pathway, thereby mediating cytoskeletal reorganization and cell movement [51]. This signaling axis represents one of the core mechanisms by which integrins regulate VSMC migration, proliferation, and differentiation [20]. Speculatively, CKAP4 may act upstream of this mechanosignaling pathway and contribute to integrin recycling, representing a potential regulatory node in this positive feedback loop.

In summary, these findings raise the possibility that bacterial OMVs may act as remote mediators, transducimg microbial signals to host vascular remodeling processes. Collectively, they offer a preliminary framework for understanding how periodontal pathogens may remotely contribute to vascular pathology and suggest that CKAP4 warrants further investigation as a potential therapeutic target in preclinical models of atherosclerosis-related vascular remodeling.

## 5. Conclusions

Given that phenotypic switching and ECM dysregulation are recognized as key cellular events in atherosclerotic plaque progression, the present findings provide preliminary clues suggesting a potential association between periodontal infection and vascular remodeling processes that may contribute to atherogenesis. Such findings may offer valuable experimental evidence elucidating the mechanisms underlying the potential link between periodontitis and atherosclerosis. Furthermore, they may imply a role for the oral microbiome in the remote regulation of CVDs, while simultaneously providing a theoretical basis for the development of targeted early interventions for CVDs.

Nevertheless, the present study has several limitations. First, the findings lack rigorous validation through in vivo models, as the current experimental system cannot fully replicate the complex vascular microenvironment, host immunity, or systemic metabolic conditions characteristic of intact organisms. The 2.5 µg/mL dose was selected empirically on the basis of VSMC viability and functional response gradients; thus, it represents a proof-of-concept concentration rather than a direct physiological equivalent. To define physiologically relevant tissue levels and further verify the underlying mechanisms in vivo, future investigations using fluorescently labeled OMVs in animal models of periodontitis-associated atherosclerosis are warranted. Moreover, given the inherent species differences between murine and human vascular cells, additional validation using human primary VSMCs is required to clarify the translational relevance of the CKAP4/ITGα5β1 signaling axis identified in MOVAS cells.

Another important limitation is that OMVs are naturally enriched with LPS, gingipains, and other pro-inflammatory components. Therefore, it is unclear whether the observed phenotypic switching, wound closure, and ECM remodeling are specifically mediated by intact OMVs or primarily driven by endotoxin-activated pathways that could also be induced by purified *P. g*-LPS alone. Our study lacked essential controls, such as heat-inactivated OMVs, proteinase K-treated OMVs, purified LPS, and OMVs from LPS-modified mutants, so the conclusion that CKAP4 is specifically involved in *P. g*-OMV-induced VSMC dysfunction remains preliminary. Future studies should include these controls to clarify OMV-specific effects.

Beyond the aforementioned issues, it remains unclear whether the observed effects are unique to *P. g*-OMVs or represent a general property of extracellular vesicles derived from other periodontal pathogens—a question that should be addressed in future comparative studies. Additionally, although we speculate that ECM stiffness may change following OMV-induced ECM remodeling, direct measurements of ECM mechanical properties and their impact on VSMC function are warranted. High-throughput approaches, such as transcriptomics and proteomics, will also help elucidate the molecular networks underlying the action of *P. g*-OMVs.

## Data Availability

The original contributions presented in this study are included in this article. Further inquiries can be directed to the corresponding author.
